# Cytotoxicity of Crude Extract and Isolated Constituents of the* Dichrostachys cinerea* Bark towards Multifactorial Drug-Resistant Cancer Cells

**DOI:** 10.1155/2019/8450158

**Published:** 2019-07-08

**Authors:** Armelle T. Mbaveng, Francois Damen, James D. Simo Mpetga, Maurice D. Awouafack, Pierre Tane, Victor Kuete, Thomas Efferth

**Affiliations:** ^1^Department of Pharmaceutical Biology, Institute of Pharmacy and Biochemistry, University of Mainz, Staudinger Weg 5, 55128 Mainz, Germany; ^2^Department of Biochemistry, Faculty of Science, University of Dschang, P.O. Box 67, Dschang, Cameroon; ^3^Department of Chemistry, Faculty of Science, University of Dschang, P.O. Box 67, Dschang, Cameroon

## Abstract

The effectiveness of anticancer chemotherapy is greatly impeded by the resistance of malignant cells to cytotoxic drugs. In this study, the cytotoxicity of the crude extract (DCB) and compounds isolated from the bark of* Dichrostachys cinerea, *namely, betulinic acid (**1**), glyceryl-1-hexacosanoate (**2**), 7-hydroxy-2-(4-hydroxyphenyl)-4*H*-chromen-4-one (**3**), and 6-hydroxy-2-(4-hydroxyphenyl)-4*H*-chromen-4-one (**4**), was investigated. The study was extended to the assessment of the mode of induction of apoptosis by DCB and compound** 1**. The resazurin reduction assay was used for cytotoxicity studies. Assessments of cell cycle distribution, apoptosis, mitochondrial membrane potential (MMP), and reactive oxygen species (ROS) were performed by flow cytometry. Constituents of DCB were isolated by column chromatography. Triterpenoid** 1** and flavone** 4** had cytotoxic effects towards the 9 tested cancer cell lines with IC_50_ values below 50 *μ*M. The recorded IC_50_ values varied from 7.65 *μ*M (towards multidrug-resistant CEM-ADR5000 leukemia cells) to 44.17 *μ*M (against HepG2 hepatocarcinoma cells) for** 1**, 18.90 *μ*M (CCRF-CEM leukemia cells) to 88.86 *μ*M (against HCT116p53^+/+^ colon adenocarcinoma cells) for** 4,** and 0.02 *μ*M (against CCRF-CEM cells) to 122.96 *μ*M (against CEM/ADR5000 cells) for doxorubicin. DCB induced apoptosis in CCRF-CEM cells mostly mediated by MMP alteration and enhanced ROS production; compound** 1** induced apoptosis through caspases activation and MMP alteration and increased ROS production.* Dichrostachys cinerea *is an interesting cytotoxic plant and deserves more studies leading to new antineoplastic agents to fight cancer and mostly leukemia.

## 1. Introduction

Recent data from the World Health Organization revealed that most countries still face an increase in cancer incidences [1]. The global cancer burden reached 18.1 million new cases in 2018, with one in eight men and one in 11 women dying in developing countries [1]. Worldwide, the five-year prevalence of cancer is estimated at 43.8 million people [1]. The effectiveness of anticancer chemotherapy is greatly impeded by the resistance of malignant cells to cytotoxic drugs [2]. The search for new antiproliferative drugs should therefore take into consideration the ability of cancer cells to develop resistant phenotypes. Natural products are well recognized as source of cytotoxic molecules [3]. Various studies have previously documented the effectiveness of botanicals and phytochemicals from the flora of Africa to fight cancer multidrug resistance (MDR) [4, 5]. However, research should be intensified to increase the library of cytotoxic plants and molecules available in the African flora, in order to have better chances of achieving clinically exploitable drugs in the future. The present study was hence designed to evaluate the cytotoxicity of crude extract and compounds from the bark of* Dichrostachys cinerea* (L.) Wight & Arn. (Fabaceae) towards a panel of drug-sensitive and drug-resistant cancer cell lines. The mode of induction of apoptosis of crude extract and compound** 1 **was further investigated.* Dichrostachys cinerea, *also known as sicklebush, Bell mimosa, Chinese lantern tree, or Kalahari Christmas tree, is a fast growing tree of up to 7 m height, traditionally used as laxative, diuretic and to treat dysentery, elephantiasis, gonorrhoea, boils, headache, syphilis, sore, worms [6, 7], inflammation, and cancer [8]. Previous phytochemical analysis of* Dichrostachys cinerea* led to the identification of a triterpenoid *β*-amyrin glucoside, apigenin-7-*O*-apiosyl (1→2) glucoside, chrysoeriol-7-*O*-apiosyl (1→2) glucoside, clovamide, quercetin-3-*O*-rhamnopyranoside, quercetin-3-*O*-glucopyranoside, myricetin-3-*O*-rhamnopyranoside, myricetin-3-*O*-glucopyranoside, myricetin, apigenin, and kaempferol from the leaves [6, 9] as well as the meroterpene derivatives, dichrostachines A-R from the bark and roots [10]. Preliminary cytotoxicity investigations of this plant were reported towards DU145 and 22Rv1 prostate cancer cells and HeLa cervical cancer cells [7]. This is the first intensive study on the potential of* Dichrostachys cinerea *and some of its constituents against MDR cancer cell lines.

## 2. Materials and Methods

### 2.1. Plant Material and Extraction

*Dichrostachys cinerea *barks were collected in February 2017 in Bazou (5° 4′ 0′′ N, 10° 28′ 0′′ E) in the West Region of Cameroon. The plant was identified at the National Herbarium of Cameroon (Yaoundé), where voucher is available under number 34028/HNC. The bark of* D. cinerea* was air-dried and powdered (2000 g) and then macerated in 20 l of ethanol for 48 h. The solvent was evaporated in vacuum under reduced pressure to give the crude extract (170 g; DCB).

### 2.2. Isolation of Compounds from the Bark of* Dichrostachys cinerea*

An aliquot of DCB (160 g) was treated with ethyl acetate (EtOAc) to give two subextracts: the EtOAc extract (DCA, 85g) and the methanol (MeOH) extract (DCB, 75g). DCA (85 g) was submitted to a silica gel flash column chromatography (CC) using dichloromethane (CH_2_Cl_2_)-EtOAc and EtOAc-MeOH mixtures of increasing polarity. Fractions of 150 ml each were collected as follows: CH_2_Cl_2_ 100% (sub-frs 1-8), CH_2_Cl_2_-EtOAc 95:5 (sub-frs 9-19), CH_2_Cl_2_-EtOAc 90:10 (sub-frs 20-23), CH_2_Cl_2_-EtOAc 80:20 (sub-frs 24-30), CH_2_Cl_2_-EtOAc 60:40 (sub-frs 31-35), CH_2_Cl_2_-EtOAc 50:50 (sub-frs 36-40), EtOAc100% (sub-frs 41-45), EtOAc- MeOH 95:20 (sub-frs 46-52), EtOAc-MeOH 90:10 (sub-frs 53-60), EtOAc-MeOH 80:20 (sub-frs 61-64), EtOAc-MeOH 70:30 (sub-frs 65-68), and MeOH 100% (sub-frs 69-72). These fractions were then pooled on the basis of their analytical thin layer chromatography (TLC) profiles into five fractions (frs) as follows: DCA1 (Sub-frs 1-6; 10 g), DCA2 (Sub-frs 7-14; 12 g), DCA3 (Sub-frs 15-30; 13 g), DCA4 (Sub-frs 31-60; 20 g), and DCA5 (Sub-frs 61-72; 25 g). From a direct filtration of fraction DCA2, followed by further Sephadex CC, compound** 1** was obtained as a white powder (1 g).

An aliquot of DCA5 (18 g) was submitted to silica gel flash CC using CH_2_Cl_2_-EtOAc and EtOAc-MeOH mixtures of increasing polarity. 110 subfractions (sub-frs) of 150 ml each were collected as follows: CH_2_Cl_2_100% (sub-frs 1-22), CH_2_Cl_2_-EtOAc 95:5 (sub-frs 23-53), CH_2_Cl_2_-EtOAc 90:10 (sub-frs 54-59), CH_2_Cl_2_-EtOAc 85:15 (sub-frs 60-75), CH_2_Cl_2_-EtOAc 80:20 (sub-frs 76-83), CH_2_Cl_2_-EtOAc 75:25 (sub-frs 84-91), CH_2_Cl_2_-EtOAc 70:30 (sub-frs 92-95), CH_2_Cl_2_-EtOAc 60:40 (sub-frs 96-100), EtOAc100% (sub-frs 101-104), EtOAc-MeOH 90:10 (sub-frs 105-107), and MeOH 100% (sub-frs 108-110). Compound** 3** was obtained as a white powder (14 mg) in sub-frs 27-31; sub-frs 30-35 afforded compound** 2** as yellow powder (15 mg); meanwhile, sub-frs 37-44 yielded compound** 4** as yellow powder (15 mg).

### 2.3. General Procedure

All general chemistry procedures (mass spectral data, ^1^H and ^13^C nuclear magnetic resonance (NMR) spectra) and CC were performed with the same apparatus and reagents, and in similar experimental conditions as reported earlier [13].

### 2.4. Cell Cultures

Drug-sensitive and drug-resistant cancer cell lines of previously reported origin were used in this study. These included drug-sensitive CCRF-CEM leukemia cells and its multidrug-resistant P-glycoprotein-overexpressing subline CEM/ADR5000 cells [14–16], MDA-MB-231-pcDNA breast cancer cells and their resistant subline MDA-MB-231-*BCRP *clone 23 cells [17], HCT116 p53^+/+^colon cancer cells and their knockout clone HCT116 p53^−/−^ cells, and U87.MG glioblastoma cells and their resistant subline U87.MGΔ*EGFR *cells [18, 19]. Normal AML12 hepatocytes were used and compared with HepG2 hepatocarcinoma cells [18, 19].

### 2.5. Cytotoxicity Assay

The cytotoxicity assay performed using resazurin reduction assay was applied to the crude extract (DCB), compounds** 1-4,** and doxorubicin [18, 20, 21] with similar experimental conditions as those reported earlier [13, 19, 22, 23]. The Infinite M2000 Pro™ plate reader (Tecan, Crailsheim, Germany) with excitation wavelength of 544 nm and an emission wavelength of 590 nm was used to read the fluorescence after 72 h incubation. IC_50_ values earlier defined [13] were calculated from a calibration curve by linear regression using Microsoft Excel [24]. The degree of resistance (D.R.) was determined as the IC_50_ value of the resistant cell line* versus* that of its sensitive congeners; meanwhile, the selectivity index (S.I.) was the IC_50_ value in normal AML12 hepatocytes* versus* that in HepG2 hepatocarcinoma.

### 2.6. Cell Cycle Analysis and Detection of Apoptotic Cells by Flow Cytometry and Annexin V/PI Staining

Aliquots of 1×10^6^ CCRF-CEM cells were treated with the studied samples (DCB and compound** 1**), the reference drug (doxorubicin), or the solvent control (DMSO) at various concentrations. The distribution of CCRF-CEM cycle was analyzed as described earlier in similar experimental conditions (24 h incubation; humidified 5% CO_2_ atmosphere; 37°C) [13, 22, 23]. The BD Accuri C6 Flow Cytometer (BD Biosciences, Heidelberg, Germany) was used to measure the propidium iodide (PI) fluorescence of individual nuclei. Assays were repeated at least three times and in triplicate.

To perform the annexin V/PI staining, DCB, betulinic acid (**1**), and doxorubicin were used to treat an amount of 1×10^6^ per 1 ml CCRF-CEM cells. The experimental conditions were similar to those earlier reported (24 h incubation; humidified 5% CO_2_ atmosphere; 37°C) [13]. The BD Accuri C6 Flow Cytometer was then used to analyze apoptosis using fluorescein isothiocyanate (FITC)-conjugated annexin V/PI assay kit (eBioscience™ Annexin V; Invitrogen, San Diego, USA) similarly as reported earlier [13, 22, 23]; early apoptosis for cells stained with only annexin V; late apoptosis or in a necrotic stage for cells stained with both annexin V and propidium iodide [13, 25, 26].

### 2.7. Assessment of Caspases Activation Using the Caspase-Glo Assay

After 6 h treatment of CCRF-CEM cells with DCB and triterpenoid** 1** for 6 h, caspases activities were evaluated with Caspase-Glo 3/7, 8, and 9 assay kits (Promega, Mannheim, Germany) similarly as previously reported [13, 18, 27].

### 2.8. Assessment of the Integrity of the Mitochondrial Membrane

The mitochondrial membrane potential (MMP) of CCRF-CEM cells was analyzed after 24 h treatment with DCB, compound** 1,** or valinomycin (mitochondrial gradient dissipation substance or positive control). The 5,5′,6,6′-tetrachloro-1,1′,3,3′-tetraethylbenzimidazolyl carbocyanine iodide (JC-1; Biomol, Hamburg, Germany) staining was used to measure the MMP similarly as previously reported [13, 18, 22, 23].

### 2.9. Evaluation of the Production of Reactive Oxygen Species (ROS)

The measurement of ROS production using 2′,7′-dichlorodihydrofluorescein diacetate (H_2_DCFH-DA) (Sigma-Aldrich) was done in CCRF-CEM cells were treated with DCB, compound** 1**, a solvent control (DMSO), or a positive control, hydrogen peroxide (H_2_O_2_) for 24 h, in similar experimental conditions as documented earlier [13, 18, 28, 29].

## 3. Results

### 3.1. Phytochemistry

**Figure 1:**
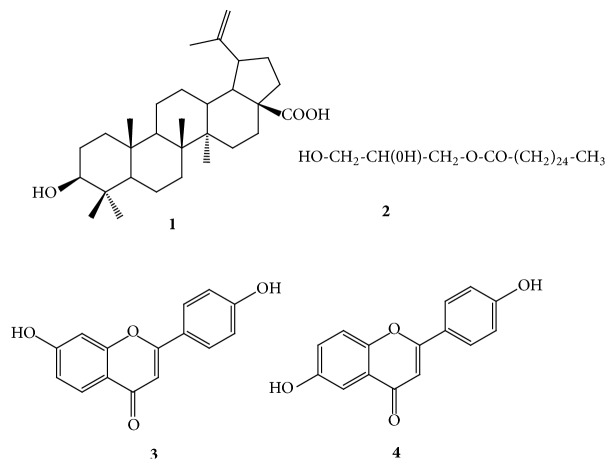
Chemical structure of compounds isolated from the bark of* Dichrostachys cinerea*.** 1**: betulinic acid;** 2**: glyceryl-1-hexacosanoate;** 3**: 7-hydroxy-2-(4-hydroxyphenyl)-4*H*-chromen-4-one; and** 4**: 6-hydroxy-2-(4-hydroxyphenyl)-4*H*-chromen-4-one.

Physical and NMR data with direct comparison with literature was used to elucidate the chemical structures of phytochemicals isolated from the bark of* Dichrostachys cinerea*. They were betulinic acid, C_30_H_50_O (**1**; m.p. 216°C;* m/z *426) [30], glyceryl-1-hexacosanoate, C_29_H_58_O_4_ (**2**; m.p. 91-93°C;* m/z *470) [31], 7-hydroxy-2-(4-hydroxyphenyl)-4*H*-chromen-4-one, C_15_H_10_O_4_ (**3**; m.p. 315°C;* m/z *254) [32], and 6-hydroxy-2-(4-hydroxyphenyl)-4*H*-chromen-4-one, C_15_H_10_O_4_ (**4**; m.p. 325°C;* m/z* 254 ) [33] (Figure 1).

### 3.2. Cytotoxicity

Triterpenoid** 1** and flavone** 4** had cytotoxic effects towards the 9 tested cancer cell lines with IC_50_ values below 50 *μ*M (Table 1). Botanical DCB and flavone** 3** had selective activities, while no cytotoxic effect (IC_50_ value above 100 *μ*M) was recorded with fatty acid ester** 2**. The recorded IC_50_ values varied from 7.65 *μ*M (towards resistant CEM-ADR5000 leukemia cells) to 44.17 *μ*M (against HepG2 hepatocarcinoma cells) for** 1**, 18.90 *μ*M (CCRF-CEM leukemia cells) to 88.86 *μ*M (against HCT116p53^+/+^ colon adenocarcinoma cells) for** 4,** and 0.02 *μ*M (against CCRF-CEM cells) to 122.96 *μ*M (against CEM/ADR5000 cells) for doxorubicin. The IC_50_ values in normal AML12 hepatocytes were above 80 *μ*g/mL for DCB and above 100 *μ*M for compounds** 2** and** 3** (Table 1). Collateral sensitivity (hypersensitivity or D.R. below 1) of all resistant cell lines compared to their sensitive counterparts was observed with triterpenoid** 1** (Table 1). Hypersensitivity or normal sensitivity of at least one resistant cell line to botanical DCB as well as compounds** 3** and** 4** was also recorded (Table 1). Selectivity indexes above 2 were also observed with compound** 1** (S.I.: >2.13) and doxorubicin (S.I.: 11.59) in HepG2 as compared with normal AML12 hepatocytes (Table 1).

### 3.3. Cell Cycle Distribution and Apoptosis

**Figure 2:**
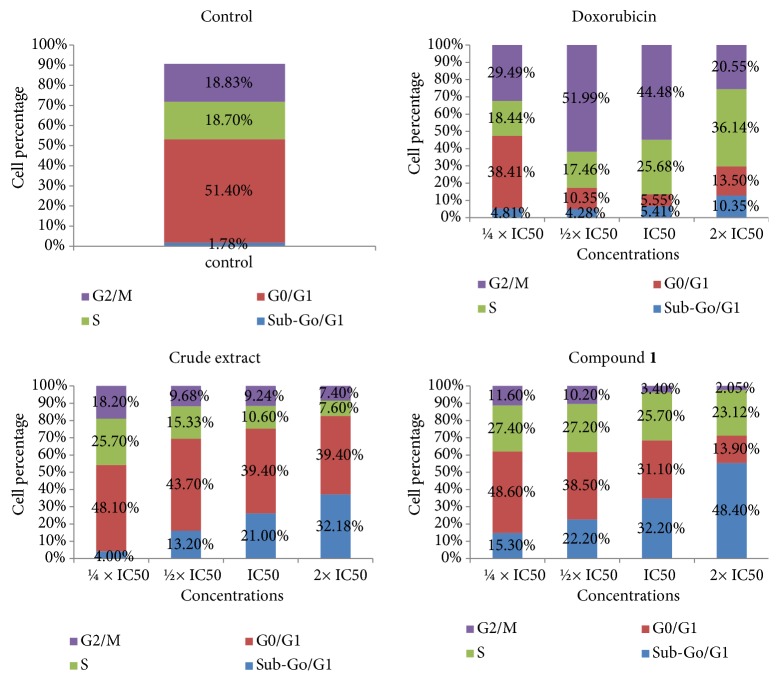
Distribution of CCRF-CEM leukemia cell cycle upon 24 h treatment with crude extract, betulinic acid (**1**), and doxorubicin. IC_50_ values were 5.69 *μ*g/ml for the crude extract, 8.80 *μ*M for** 1,** and 0.02 *μ*M for doxorubicin.

**Figure 3:**
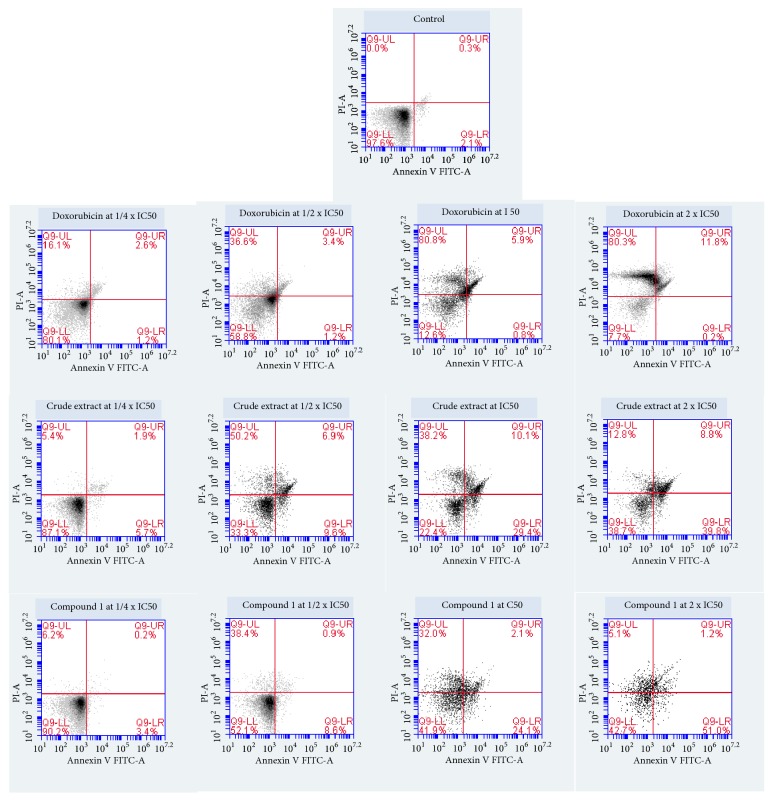
Evaluation of apoptosis induced by the crude extract, betulinic acid (**1**), and doxorubicin on CCRF-CEM leukemia cells after 24 h as determined by annexin V/PI assay. Apoptosis was assessed by flow cytometry after annexin V-PI double staining. IC_50_ values were 5.69 *μ*g/mL for the crude extract, 8.80 *μ*M for** 1,** and 0.02 *μ*M for doxorubicin. Necrotic cells lose membrane integrity, allowing PI entry. Q9-LL: viable cells exhibit annexin V (-)/PI (-); Q9-LR: early apoptotic cells exhibit annexin (+)/PI (-); and Q9-UR and Q9-UL: late apoptotic cells or necrotic cells exhibit annexin V (+)/PI (+) or annexin V (-)/PI (+).

Upon treatment of CCRF-CEM cells with botanical DCB, triterpenoid** 1,** and the reference compound doxorubicin, the cell cycle phases were modified in concentration-dependent manner (Figure 2). Increase of cells in sub-G0/G1 phase was observed with all samples, and DCB induced cell cycle arrest in G0/G1 phase, while triterpenoid** 1** caused cycle arrest in G2/M; doxorubicin induced arrest of cell cycle between S and G2/M. The percentage of CCRF-CEM cells in sub-G0/G1 phase in nontreated cells only was 1.78%; meanwhile, it varied upon treatment from 4.00% (1/4 × IC_50_) to 32.18% (2 × IC_50_) for DCB, 15.30% (1/4 × IC_50_) to 48.40% (2 × IC_50_) for compound** 1,** and 4.81% (1/4 × IC_50_) to 10.35% (2 × IC_50_) for doxorubicin (Figure 2). These data suggested that DCB, compound** 1,** and doxorubicin induced apoptosis in CCRF-CEM cells. In the annexin V/PI staining, the induction of apoptosis was further investigated. The results depicted in Figure 3 showed a dose-dependent induction with DCB, triterpenoid** 1,** and doxorubicin. When cells were treated with 2 × IC_50_, for example, DCB induced apoptosis with 39.8% early apoptotic V (+)/PI (-) cells, 8.8% late apoptotic V (+)/PI (+) cells as well as necrosis with 12.8% annexin V (-)/PI (+) cells; triterpenoid** 1** induced 51.0% early apoptotic cells and 5.1% necrotic cells, while doxorubicin induced 11.8% late apoptotic cells.

### 3.4. Activation of Caspases, Integrity of MMP, and ROS Production

**Figure 4:**
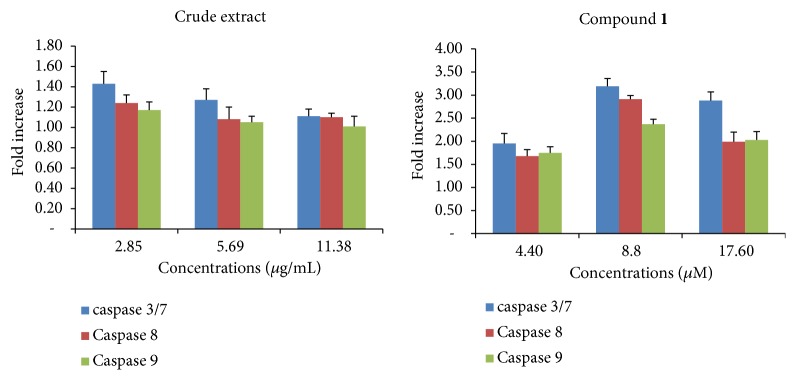
Effects of 6 h treatment of CCRF-CEM cells with crude extract and betulinic acid (**1**) on caspases activity. Samples were tested at their 1/2 × IC_50_, IC_50_ and 2 × IC_50_; IC_50_ values were 5.69 *μ*g/mL for the crude extract and 8.80 *μ*M for** 1**. Caspase activity is expressed as percentage (%) compared to untreated cells. Shown are mean±SD of three independent experiments.

Treatment of CCRF-CEM cells with DCB did not activate the activity of caspases 3/7, 8, and 9 contrary to triterpenoid** 1** (Figure 4). In effect, a dose-dependent activation of caspases upon treatment with** 1** was observed, with optimal effects at 8.8 *μ*M; up to 3.19-fold, 2.91-fold, and 2.37-fold increases in the activity of caspases 3/7, 8, and 9, respectively, were recorded.

**Figure 5:**
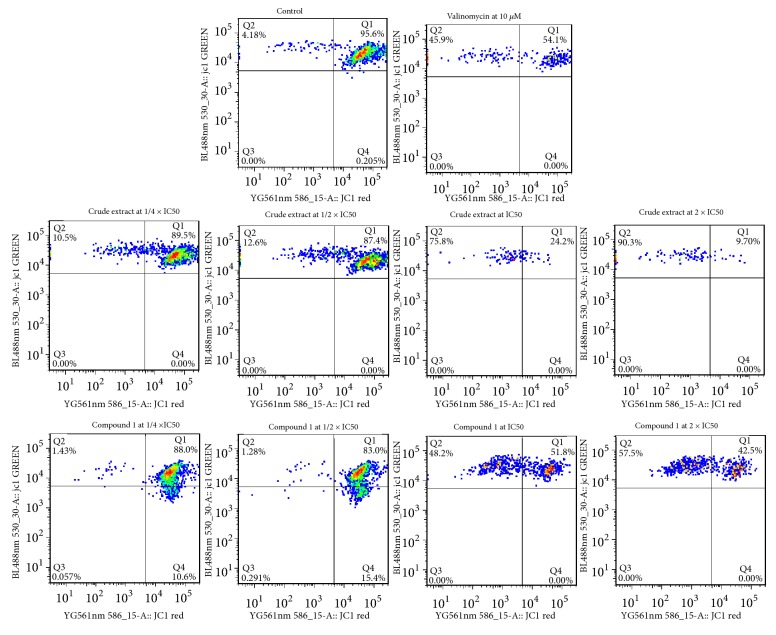
Effect of the crude extract, betulinic acid (**1**) and valinomycin for 24 h on the MMP of CCRF-CEM cells. IC_50_ values were 5.69 *μ*g/ml for the crude extract, 8.80 *μ*M for** 1,** and 0.02 *μ*M for doxorubicin. Intact cells (Q1), loss of MMP (Q2), and ruptured cell membrane (Q3 and Q4).

The effects of DCB, betulinic acid (**1**), and valinomycin on integrity of MMP in CCRF-CEM are depicted in Figure 5. Both DCB and compound** 1** considerably modified the MMP with up to 90.3% and 57.5% (at 2 × IC_50_), respectively; valinomycin at 10 *μ*M induced 45.9% alteration.

**Figure 6:**
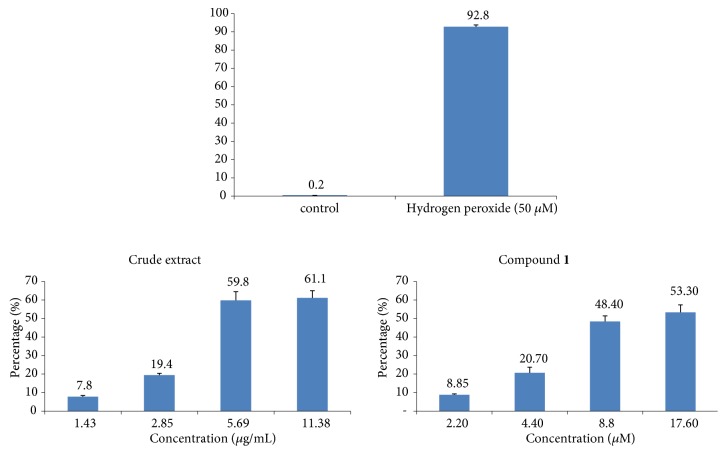
ROS production in CCRF-CEM cells treated for 24 h with the crude extract, betulinic acid (**1**), and hydrogen peroxide (H_2_O_2_). Samples were tested at their 1/4 × IC_50_, 1/2 × IC_50_, IC_50_, and 2 × IC_50_; IC_50_ values were 5.69 *μ*g/mL for the crude extract and 8.80 *μ*M for** 1**. Shown are mean±SD of three independent experiments.

The effects of DCB and compound** 1** on the production of ROS in CCRF-CEM cells are given in Figure 6. The two samples dose-dependently enhanced the production of ROS in CCRF-CEM cells. The ROS level in nontreated cells was 0.2%, whilst at 2 × IC_50_, DCB caused increased ROS production by up to 61.1% and triterpenoid** 1** by 53.30%. H_2_O_2_ induced ROS production by 98.8% at 50 *μ*M.

## 4. Discussion

Phytochemicals isolated from the bark of* Dichrostachys cinerea *were one triterpenoid** 1**, one ester of fatty acid** 2,** and two flavone-type flavonoids** 3** and** 4**. Previous phytochemical investigation of the bark of* Dichrostachys cinerea* led to the isolation of meroterpene derivatives, dichrostachines A-R [10] which were not isolated in this study, probably due to the isolation procedure used or the fact that the plant was harvested in different geographic locations.

Drug resistance of malignant cells seriously hampers the chemotherapy of cancer. In the search for cytotoxic compounds, scientists should take into consideration the ability of these cells to rapidly develop drug resistance. This is possible when investigations also consider resistant phenotypes of malignant cells. In the present study, we have used several models of MDR cancer cell lines including ATP-binding cassette (ABC)-transporter-overexpressing MDR-mediating P-glycoprotein (P-gp; ABCB1/MDR1) or breast cancer resistance protein (ABCG2/BCRP), a p53 knockout cell line, and a mutation-activated EGFR gene (ΔEGFR) cell line. The resistant P-gp overexpressing CEM/ADR5000 cells treated with the crude extract DCB were collaterally sensitive [5] compared to their sensitive parental subline CCRF-CEM cells (Table 1). Hypersensitivity of all resistant cell lines to betulinic acid as compared to their respective sensitive counterparts was also observed; for flavones** 3** and** 4**, the hypersensitivity or otherwise normally sensitive (D.R. below or around 1) of at least three resistant cell lines was also recorded. Generally, the D.Rs. recorded upon treatments with DCB, compounds** 1**,** 3,** and** 4** were lower than with doxorubicin (Table 1). Previous studies also reported the hypersensitivity of CEM/ADR5000 leukemia cells to compound** 1** as compared to its sensitive congener CCRF-CEM cells [34]. These data are indications that* Dichrostachys cinerea *and its constituents have the potential to combat cancer multidrug resistance. According to the National Cancer Institute USA (NCI), good botanicals should exert their cytotoxicity with IC_50_ values below 20 *μ*g/ml upon 48 h or 72 h incubation [11], while this set point is 10 *μ*M for phytochemicals [11, 12]. Also, NCI recommends that botanicals yielding IC_50_ values below or around 30 *μ*g/ml should undergo purification to isolate cytotoxic molecules [35]. In this work, IC_50_ values as low as 4.69 *μ*g/ml and 4.13 *μ*g/ml were recorded with the crude extract DCB, on both sensitive and resistant leukemia cells, respectively (Table 1). Selective and lower IC_50_ values were recorded with DCB on carcinoma cells, clearly indicating that this plant could likely be used to combat leukemia. This was also the case with betulinic acid (**1**), as IC_50 _values below 10 *μ*M were also recorded towards leukemia cells, and higher values obtained in carcinoma cells. Though flavones** 3** and** 4** had cytotoxic effects in several cell lines including leukemia and carcinoma phenotypes, all IC_50_ values obtained were above 10 *μ*M. This confirms the hypothesis that this plant and its constituents could mostly be used in the fight against leukemia. The good S.I. (>2) of compound** 1** also indicates that it can be used in chemotherapy (Table 1). In effect, the low cytotoxicity of betulinic acid towards the normal PBL peripheral blood lymphoblast was also reported [36]. However, its lower S.I. as compared to that of doxorubicin, clinically associated with many adverse effects to patients (despite higher S.I.), clearly indicates that further studies on the toxicity of this compound as well as the crude extract will be necessary.

To the best of our knowledge, this is the first intensive study on cytotoxicity of* Dichrostachys cinerea *and its constituents** 3** and** 4** against MDR cancer cell lines. However, preliminary antiproliferative effects of this plant were reported towards DU145 and 22Rv1 prostate cancer cells and HeLa cervical cancer cells, with the lowest IC_50_ values of 8.04 *μ*g/ml recorded in 22Rv1 cells [7]. Also, betulinic acid is a well-known cytotoxic compound [34]. Its effects have been reported towards several cancer cell lines including sensitive and resistant phenotypes such as CCRF-CEM cells and CEM/ADR5000 leukemia cells, MDA-MB-231-pcDNA and MDA-MB-231/*BCRP* breast adenocarcinoma cells, HEK293 and HEK293/*ABCB5 *embryonic kidney cells, and U87.MG and U87.MG*ΔEGFR *glioblastoma cells with IC_50_ values ranging from 15.1 *μ*M (against HEK293 cells) to 29.4 *μ*M (towards CCRF-CEM cells) [34, 36].

In this study, the crude extract DCB and triterpenoid** 1** had the best cytotoxic effects on the two leukemia cells with IC_50_ values below 10 *μ*M. They were consequently selected for further cellular mechanistic studies towards CCRF-CEM cells, such as induction of apoptosis, caspases activation, and alteration of MMP as well as the production of ROS [37]. DCB and compound** 1** induced apoptosis in CCRF-CEM cells (Figures 2 and 3). Induction of apoptosis by DCB was mediated by MMP alteration and increased ROS production, while that induced by triterpenoid** 1** was mediated by caspases activation (Figure 4), MMP alteration (Figure 5), and increased ROS production (Figure 6). Previous studies on the molecular mechanism of the cytotoxic action of compound** 1** showed that it inhibited P-gp, BCRP, and ABCB5 and mutation activated EGFR overexpressing cells. Besides, various genes significantly correlated to its activity on cell cycle regulation, microtubule formation, signal transduction, transcriptional regulation, chromatin remodeling, cell adhesion, tumor suppression, ubiquitination, and proteasome degradation [34].

## 5. Conclusions

The present study indicated that* Dichrostachys cinerea *is a potential cytotoxic plant and should be further explored to develop new antineoplastic agents to fight recalcitrant cancers. The crude extract DCB induced apoptosis in CCRF-CEM cells mostly mediated by MMP alteration and enhanced ROS production; compound** 1** induced apoptosis through caspases activation and MMP alteration and increased ROS production.

## Figures and Tables

**Table 1:** Cytotoxicity of crude extract, compounds isolated from *Dichrostachys cinerea *and doxorubicin in multifactorial drug-sensitive and -resistant cancer cells lines and normal cells following the resazurin assay with 72 h incubation.

Cell lines	Samples, IC_50_values and degrees of resistance*∗* or selectivity index*∗∗* (in bracket)					
	Crude extract^a^	Isolated compound^b^				Doxorubicin^b^
	DCB	**1**	**2**	**3**	**4**	
CCRF-CEM	**5.69 ± 1.34**	**8.80 ± 0.35**	>100	31.18 ± 0.98	18.90 ± 1.54	**0.02±0.00**
CEM/ADR5000 Degree of resistance*∗*	**4.13 ± 0.71** (0.73)	**7.65 ± 1.46** (0.87)	>100	124.21 ± 20.16 (3.98)	38.82 ± 2.13 (2.05)	122.96±10.94 (6,683.00)
MDA-MB-231-*pcDNA*	44.72 ± 2.05	38.83 ± 0.94	>100	75.55 ± 5.16	45.75 ± 4.76	**0.13±0.01**
MDA-MB-231-*BCRP* Degree of resistance	64.03 ± 2.72 (1.43)	24.91 ± 1.2 (0.64)	>100	80.00 ± 1.61 (1.06)	40.46 ± 3.90 (0.88)	**0.79±0.08** (6.14)
HCT116(*p53*^*+/+*^)	>80	31.46 ± 0.49	>100	>100	48.86 ± 5.35	**0.48±0.06**
HCT116(*p53*^*-/-*^) Degree of resistance	70.37 ±9.14 (<0.87)	17.07 ± 0.70 (0.54)	>100	66.30 ± 5.67 (<0.66)	48.62 ± 3.82 (1.00)	**1.78±0.08** (3.73)
U87MG	>80	24.91 ± 0.33	>100	61.30 ± 5.87	43.86 ± 8.19	**0.26±0.03**
U87MG.Δ*EGFR* Degree of resistance	54.65 ± 6.26 (<0.68)	13.92 ± 1.06 (0.56)	>100	53.78 ± 6.10 (0.88)	44.57 ± 2.56 (1.02)	**0.98±0.07** (3.79)
HepG2	>80	44.17± 3.15	>100	48.58 ± 7.09	34.02 ± 2.95	**4.56±0.48**
AML12 Selectivity index*∗∗*	>80	>93.90 (>2.13)	>100	>100	53.94 ± 11.22 (1.59)	52.90±4.09 (11.59)

(*∗*): the degree of resistance was determined as the ratio of IC_50_ value in the resistant divided by the IC_50_ in the sensitive cell line; CEM/ADR5000, MDA-MB-231-*BCRP, *HCT116 (*p53*^*-/-*^), and U87MG.Δ*EGFR *were used as the corresponding resistant counterparts for CCRF-CEM, MDA-MB-231-*pcDNA*, HCT116 (*p53*^*+/+*^), and U87MG, respectively; (*∗∗*): the selectivity index was determined as the ratio of IC_50_ value in the normal AML12 hepatocytes divided by the IC_50_ in HepG2 hepatocarcinoma cells. In bold: significant cytotoxic effect [4, 11, 12]; (^a^): values in *μ*g/mL; (^b^): values in *μ*M; (nd): not determined;**1: **betulinic acid; **2: **glyceryl-1-hexacosanoate; **3: **7-hydroxy-2-(4-hydroxyphenyl)-4*H*-chromen-4-one; and** 4: **6-hydroxy-2-(4-hydroxyphenyl)-4*H*-chromen-4-one.

## Data Availability

The data used to support the findings of this study are included within the article.

## Abbreviations

**1**: Betulinic acid
**2**: Glyceryl-1-hexacosanoate
**3**: 7-Hydroxy-2-(4-hydroxyphenyl)-4*H*-chromen-4-one
**4**: 6-Hydroxy-2-(4-hydroxyphenyl)-4*H*-chromen-4-one
ABC: ATP-binding cassette
BCRP: Breast cancer resistance protein
CC: Column chromatography
CH_2_Cl_2_: Dichloromethane
DCB: Crude extract from the bark of* Dichrostachys cinerea*
DMSO: Dimethylsulfoxide
D.R.: Degree of resistance
EGFR: Epidermal growth factor receptor
ESI-MS: Electrospray ionization mass spectrometry
EtOAc: Ethyl acetate
FITC: Fluorescein isothiocyanate
H_2_O_2_: Hydrogen peroxide
H2DCFH-DA: 2′,7′-Dichlorodihydrofluoresceine diacetate
JC-1: 5,5′,6,6′-Tetrachloro-1,1′,3,3′-tetraethylbenzimidazolylcarbocyanine iodide
IC_50_: 50% inhibitory concentration
MDR: Multidrug resistance
MeOH: Methanol
MMP: Mitochondrial membrane potential
NMR: Nuclear magnetic resonance
P-gp: P-glycoprotein
PI: Propidium iodide
ROS: Reactive oxygen species
Sub-frs: Subfractions
TLC: Thin layer chromatography
TMS: Tetramethylsilane.

## Supplementary Materials

Supplementary Materials
*Supplementary file.docx*. RMN ^1^H, 13C and major chemical shifts of studied compounds, betulinic acid (**1**), glyceryl-1-hexacosanoate (**2**), 7-hydroxy-2-(4-hydroxyphenyl)-4*H*-chromen-4-one (**3**), and 6-hydroxy-2-(4-hydroxyphenyl)-4*H*-chromen-4-one (**4**).
