# Supplementary material for: Cytotoxicity of Crude Extract and Isolated Constituents of the Dichrostachys cinerea Bark towards Multifactorial Drug-Resistant Cancer Cells
Source: Evid Based Complement Alternat Med. 2019 Jul 8;2019:8450158. doi: 10.1155/2019/8450158 (PMC6644236; doi:10.1155/2019/8450158)
Supplement: Supplementary Materials — Supplementary file.docx. RMN 1H, 13C and major chemical shifts of studied compounds, betulinic acid (1), glyceryl-1-hexacosanoate (2), 7-hydroxy-2-(4-hydroxyphenyl)-4H-chromen-4-one (3), and 6-hydroxy-2-(4-hydroxyphenyl)-4H-chromen-4-one (4). [file 8450158.f1.docx]

**Cytotoxicity of crude extract and isolated constituents of the *Dichrostachys cinerea* bark towards multi-factorial drug resistant cancer cells**

Armelle T. Mbaveng^a,b^, Francois Damen^c^, James D. Simo Mpetga^c^, Maurice D. Awouafack^c^, Pierre Tane^c^, Victor Kuete^a,b*^, Thomas Efferth^a^**

*^a^Department of Pharmaceutical Biology, Institute of Pharmacy and Biochemistry, University of Mainz, Staudinger Weg 5, 55128 Mainz, Germany;*

*^b^Department of Biochemistry, Faculty of Science, University of Dschang, P.O. Box 67, Dschang, Cameroon;*

*^c^ Department of Chemistry, Faculty of Science, University of Dschang, P.O. Box 67, Dschang, Cameroon*

*****Corresponding author:**

*Tel: (+237) 677355927; E-mail:* [*kuetevictor@yahoo.fr*](mailto:kuetevictor@yahoo.fr)*; P.O. Box 1499 Bafoussam, Cameroon (Prof. Dr. Victor Kuete)*

*Tel: (+49) 6131-3925751; Fax: (+49) 49-6131-3923752; E-mail:* [*efferth@uni-mainz.de*](mailto:efferth@uni-mainz.de)*; 55128 Mainz, Germany (Prof. Dr. Thomas Efferth)*

*Betulenic acid* (**1**). White powder, ^13^C NMR(125 MHz*, DMSO-d*_6_):δ177.67(C-28), 150.73(C-20), 110.09 (C-29), 77.20(C-3), 55.84 (C-17), 55.32 (C-5), 50.35(C-9), 48.95 (C-19), 47.03(C-18),42.43 (C-14), 40.69 (C-8), 38.93 (C-4), 38.69 (C-1), 38.06 (C-13), 37.15(C-10), 36.74 (C-22), 34.34(C-7), 32.14(C-16), 30.52(C-15), 29.64 (C-21) 28.54 (C-23), 27.60 (C-2), 25.47 (C-12), 20.85 (C-30), 19.37(C-6), 18.39 (C-), 16.30(C-26), 16.25(C-25), 16.16(C-24), 14.82 (C-27)


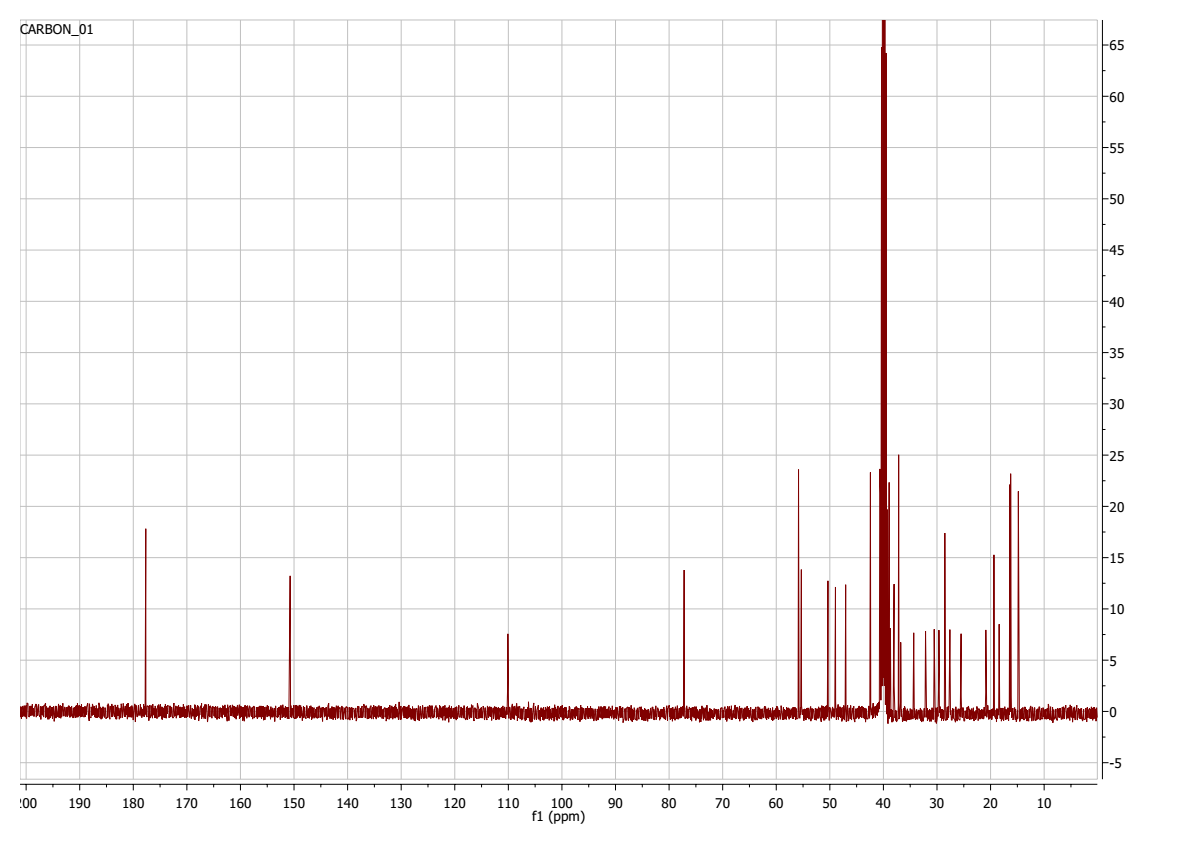


^13^C NMR (125 MHz, *DMSO-d*_6_) of compound**1**


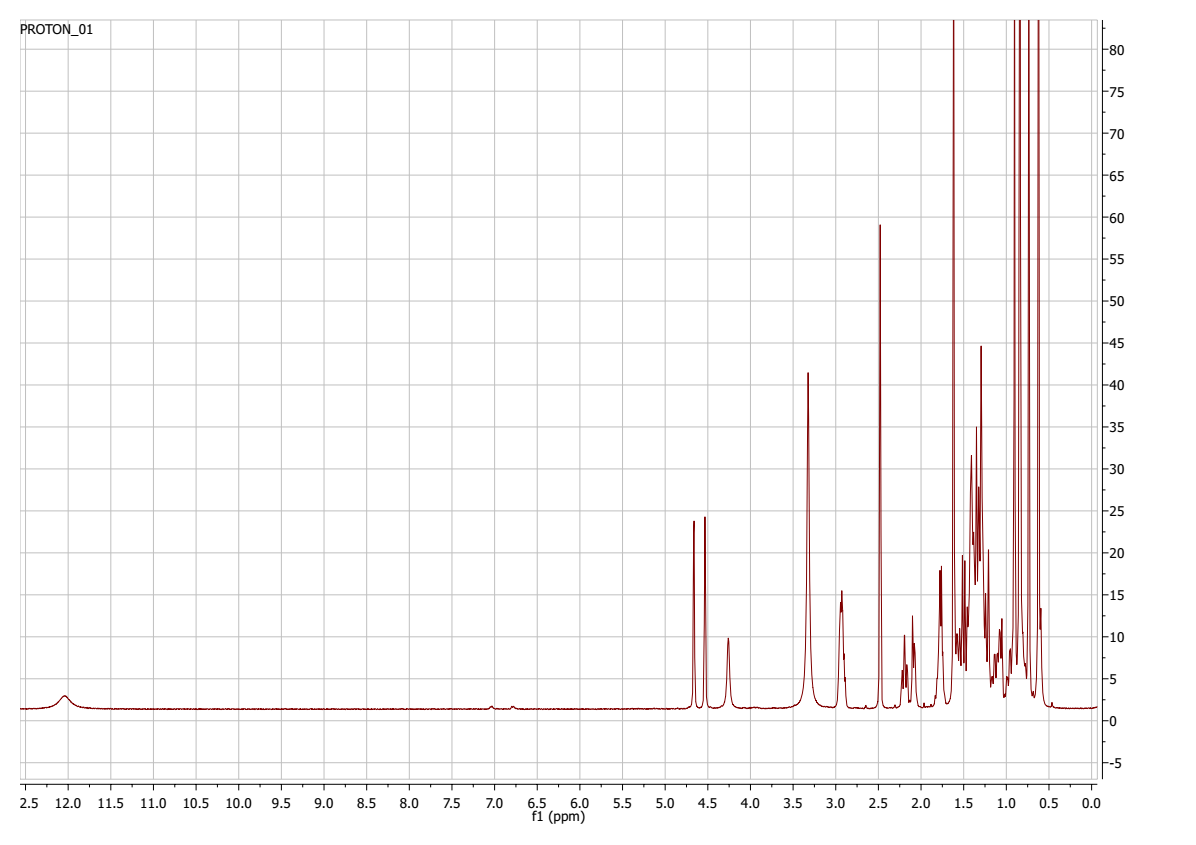


^1^H NMR (500 MHz, *DMSO-d*_6_) of compound**1**

Glycéryl-1-hexacosanoate(**2**). White powder, ^13^C NMR(125 MHz*, C_5_D_5_N*):δ173.55(C-1’), 70.72(C-2), 66.56 (C-1), 64.09(C-3), 34.23 (C-2’), 31.97-29.23(C-4’-C-24’), 25.13 (C-3’), 22.78(C-25’), 14.12 (C-26’)


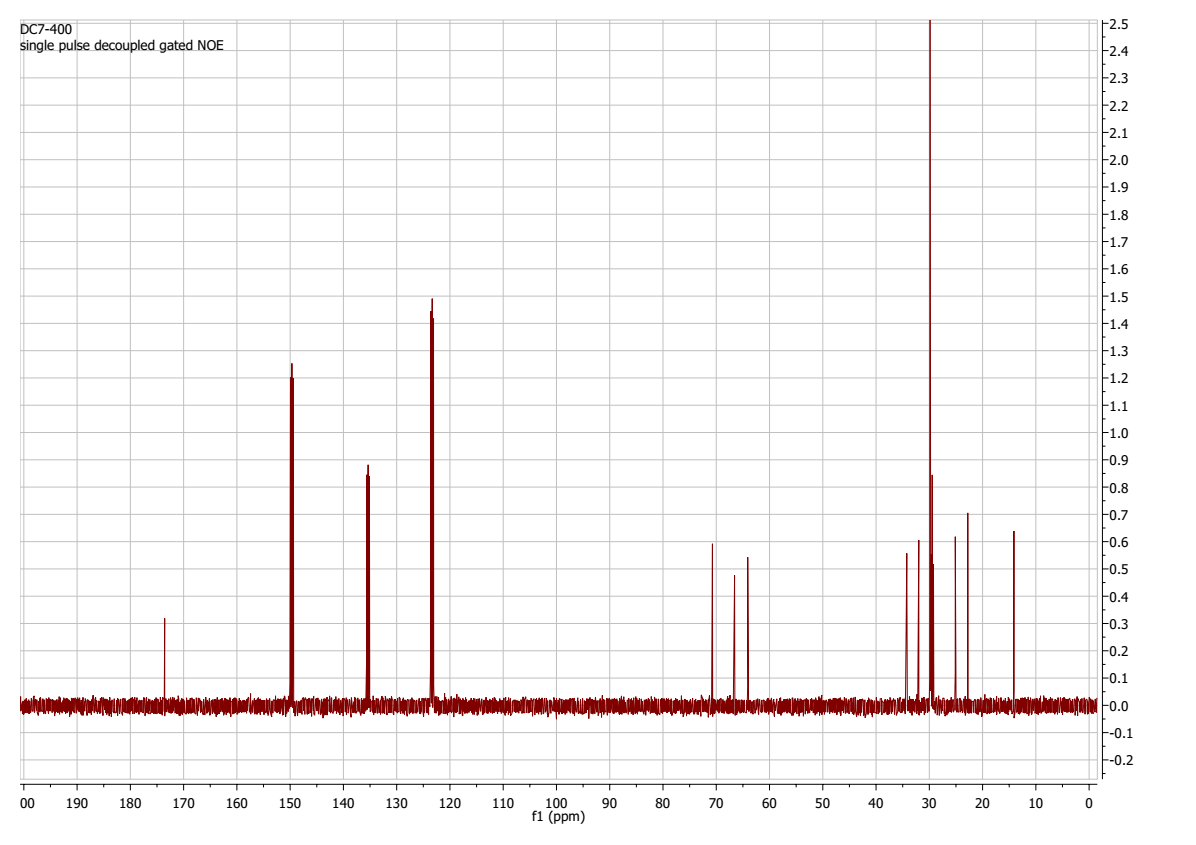


^13^C NMR (125 MHz, *C_5_D_5_N*) of compound**2**


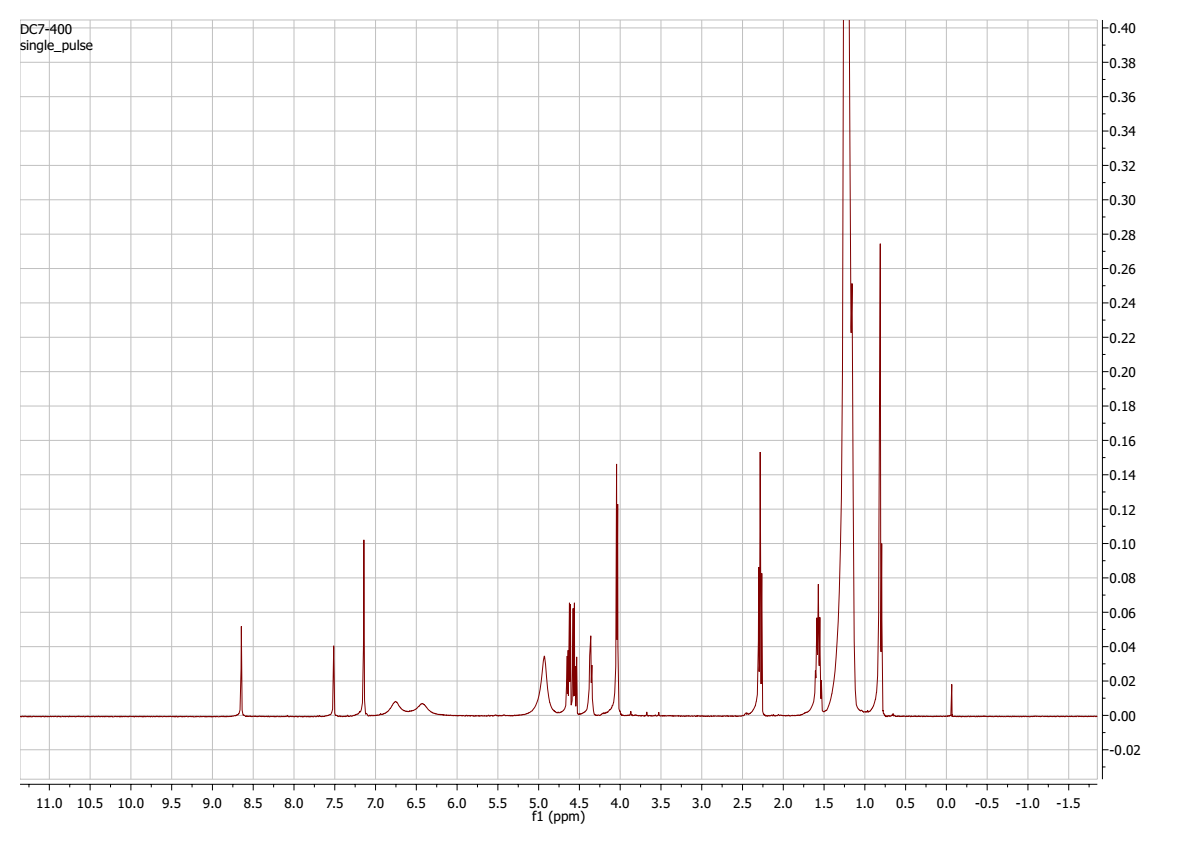


^1^H NMR (500 MHz,*C_5_D_5_N*)of compound**2**

7-Hydroxy-2-(4-hydroxyphenyl)-4H-chromen-4-one (**3**) C_15_H_10_O_4_; (mp 315°C; *m/z 254*). Yellowpowder, ^13^C NMR(125 MHz*, DMSO-d*_6_):δ176.80(C-4), 163.07(C-2), 162.97 (C-7), 161.21(C-4’), 157.90 (C-9), 128.64 (C-2’/C-6’), 126.98(C-5), 122.30 (C-1’), 116.63(C-7), 116.42 (C-3’/C-5’), 115.30 (C-10), 105.00 (C-3), 102.99 (C-8)


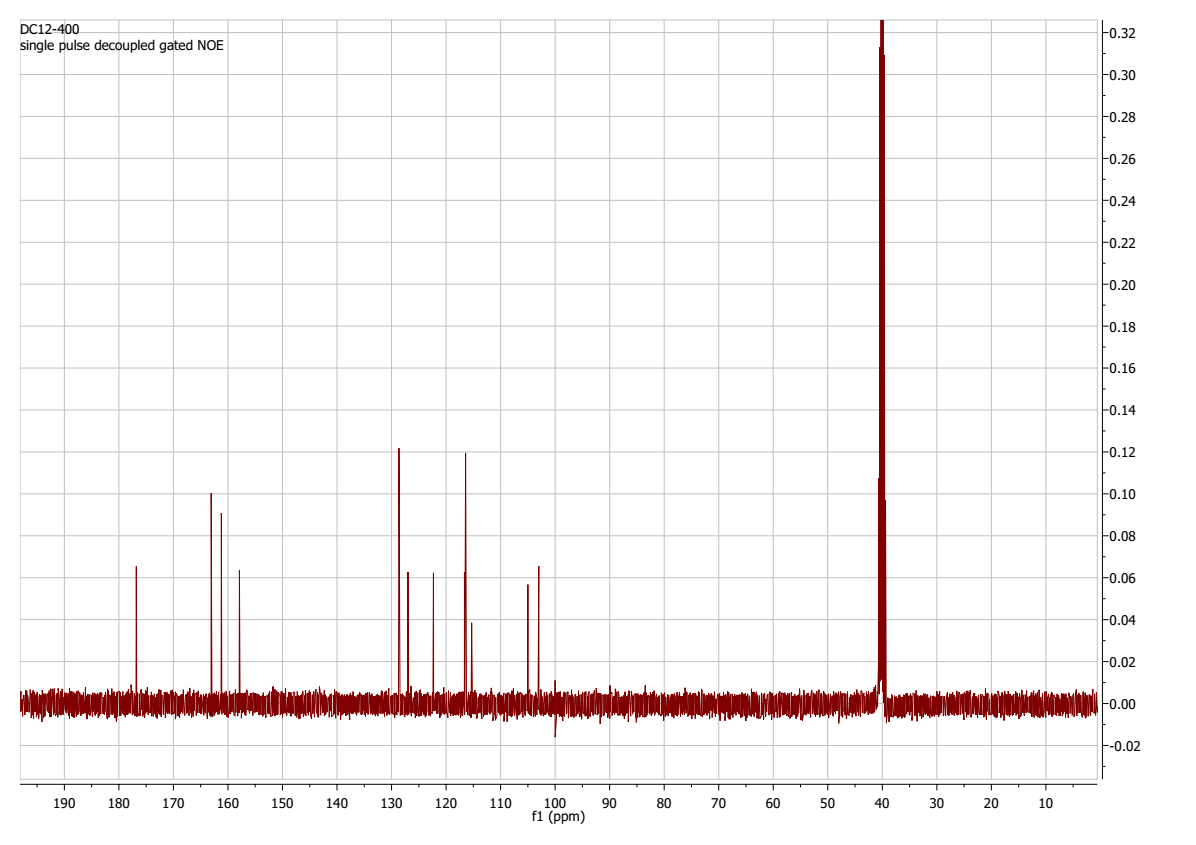


^13^C NMR (125 MHz, *DMSO-d*_6_) of **3**


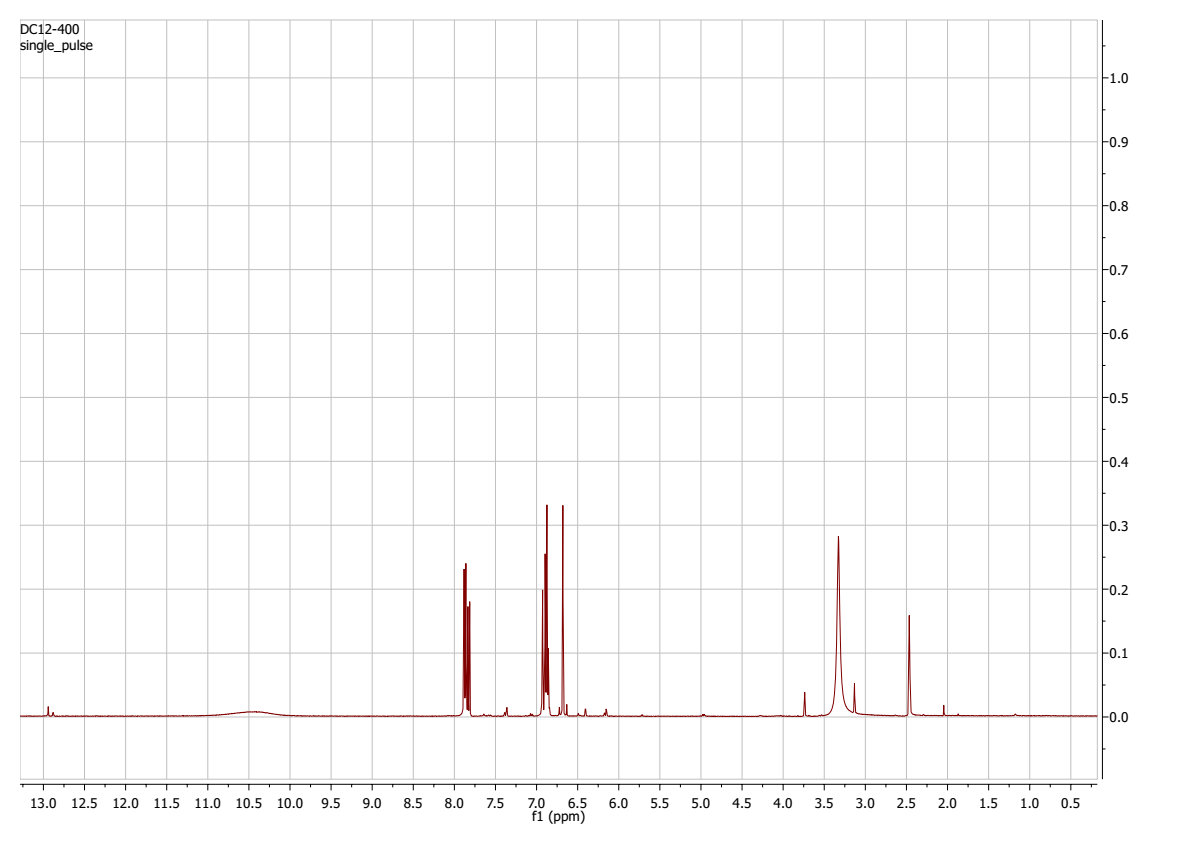


^1^H NMR (500 MHz, *DMSO-d*_6_) of compound**3**

6-Hydroxy-2-(4-hydroxyphenyl)-4H-chromen-4-one (**4**) C_15_H_10_O_4_; (mp325 ^o^C; 5*m/z* 254). White powder, ^13^C NMR(125 MHz*, C_5_D_5_N*):δ177.21(C-4), 163.98(C-2), 163.17 (C-4’), 162.04 (C-6), 158.42 (C-9), 128.47 (C-2’/C-6’), 127.28(C-10), 122.83 (C-7), 117.28(C-1’), 116.61 (C-3’/C-5’), 115.36 (C-5’), 105.62 (C-5), 103.29 (C-3)


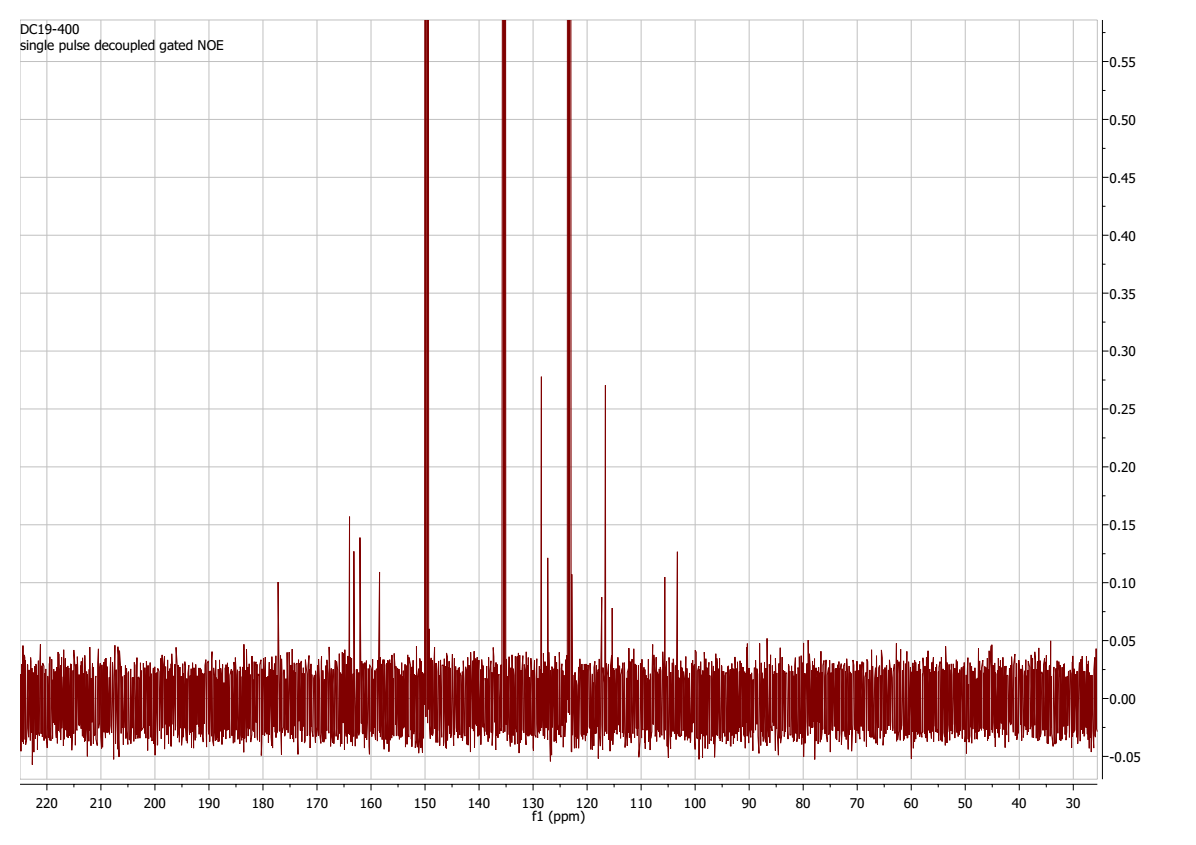


^13^C NMR (125 MHz, *C_5_D_5_N*) of compound**4**


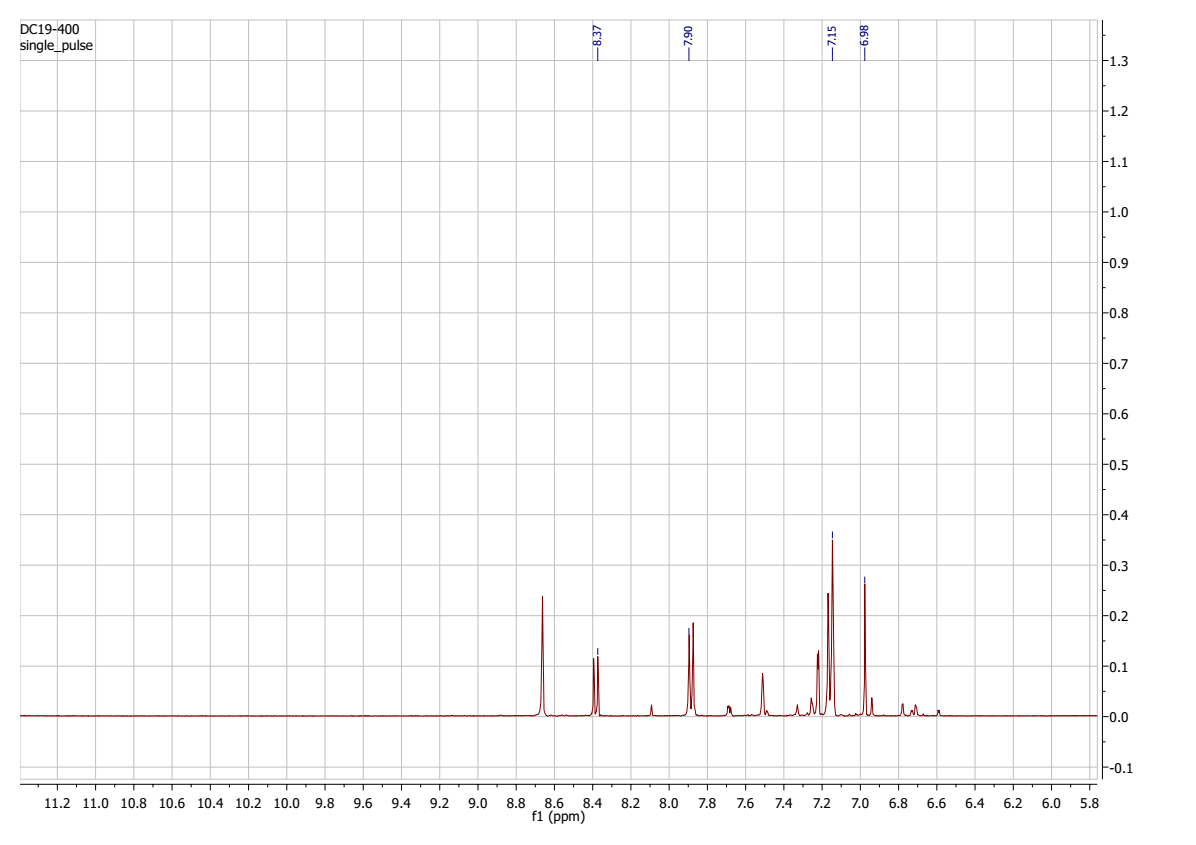


^1^H NMR (500 MHz, *C_5_D_5_N*) of compound**4**
